# Estimation of land-surface evaporation at four forest sites across Japan with the new nonlinear complementary method

**DOI:** 10.1038/s41598-017-17473-0

**Published:** 2017-12-19

**Authors:** Zhipin Ai, Qinxue Wang, Yonghui Yang, Kiril Manevski, Xin Zhao, Deni Eer

**Affiliations:** 1 Center for Regional Environmental Research, National Institute for Environmental Studies, Tsukuba, 305-8506 Japan; 2 Key Laboratory of Agricultural Water Resources, Center for Agricultural Resources Research, Institute of Genetics and Developmental Biology, Chinese Academy of Sciences, Shijiazhuang, 050021 China; 3 Department of Agroecology, Aarhus University, Tjele, 8830 Denmark; 4 Sino-Danish Center for Education and Research, Beijing, 100190 China; 5 Key Laboratory of Ecosystem Network Observation and Modeling, Institute of Geographic Sciences and Natural Resources Research, Chinese Academy of Sciences, Beijing, 100101 China

## Abstract

Evaporation from land surfaces is a critical component of the Earth water cycle and of water management strategies. The complementary method originally proposed by Bouchet, which describes a linear relation between actual evaporation (E), potential evaporation (E_po_) and apparent potential evaporation (E_pa_) based on routinely measured weather data, is one of the various methods for evaporation calculation. This study evaluated the reformulated version of the original method, as proposed by Brutsaert, for forest land cover in Japan. The new complementary method is nonlinear and based on boundary conditions with strictly physical considerations. The only unknown parameter (*α*_e_) was for the first time determined for various forest covers located from north to south across Japan. The values of *α*_e_ ranged from 0.94 to 1.10, with a mean value of 1.01. Furthermore, the calculated evaporation with the new method showed a good fit with the eddy-covariance measured values, with a determination coefficient of 0.78 and a mean bias of 4%. Evaluation results revealed that the new nonlinear complementary relation performs better than the original linear relation in describing the relationship between E/E_pa_ and E_po_/E_pa_, and also in depicting the asymmetry variation between E_pa_/E_po_ and E/E_po_.

## Introduction

Evaporation, i.e., the transfer of moisture from the surface to the atmosphere, is a critical component of the land surface water and energy balances for many Earth systems. One of the various methods for estimation of land surface evaporation is the complementary method originally described by Bouchet^1,2^. This method establishes a linear and complementary relationship between actual evaporation (E), potential evaporation (E_po_), and apparent potential evaporation (E_pa_), where the latter two can be estimated from routinely measured weather data. Various studies have utilized the Bouchet’s complementary method. Notably, Morton^3^ used the Priestley–Taylor equation^4^ to estimate E_po_, and a modified Penman’s equation^5^ to estimate E_pa_. Combined with these studies, Brutsaert and Stricker^6^ extended the formulations by the original Penman equation^7^ and the two different forms of wind function^7,8^ and proposed the famous advection-aridity model. Later on, Brutsaert and Parlange^9^ extended the Bouchet’s complementary method by introducing two parameters in order to explain the “evaporation paradox”, i.e. the decrease in evaporation measured in the past few decades over large areas with different climates.

The above-mentioned investigations are based on linear complementary relationship. By introducing zero and first-order boundary conditions, Han^10^ put forward a nonlinear equation of the complementary relationship. Recently, Brutsaert^11^ pointed on the limitations and errors in the equation developed by Han^10^, and proposed an improved new nonlinear equation and its application in combination with the advection-aridity method^12^. Few recent studies have been conducted to assess the performance of this new nonlinear generalization for sites in Australia and China^12–14^. However, as indicated by Zhang^13^, the new method is yet to be widely applied and evaluated among different climate zones and land cover types.

Forest area is reported to be 68.5% of total land area of Japan, according to the World Bank data for 2015. Forests are part of the natural and cultural fabric of Japan, one of the most densely populated countries in the world that is also heavily industrialized. Therefore, a better understanding of forest evaporation plays an important role in better understanding the water cycle and the corresponding water resources management for the forests in Japan. The objective of this study is to implement and evaluate the new nonlinear complementary relationship for estimation of land surface evaporation at four forest sites located from north to south across Japan. The parameter *α*_e_, a critical unknown coefficient in the new nonlinear method, was determined for the first time for different types of forest in Japan. As emphasized by Brutsaert^12^, this study demonstrates the performance and validity of the new nonlinear complementary method for the forest ecosystem among different climate zones, which facilitates its application in future hydrological studies.

## Result

### Determined α_e_

The specific values of *α*_e_ and associated coefficient of determination (R^2^) for each year and site are listed in Table 1. As seen from the table, all values of *α*_e_ were close to 1. Mean *α*_e_ value was 1.04 (R^2^ = 0.61), 1.03 (R^2^ = 0.81), 1.02 (R^2^ = 0.82), and 0.95 (R^2^ = 0.87) for Sapporo, Kawagoe, Fujiyoshida and Kahoku site, respectively, whereas its corresponding range was 1.03–1.05, 0.98–1.10, 0.99–1.05, and 0.94–0.95. Thus, the highest *α*_e_ value was found at Kawagoe site in 2000, whereas the lowest value was found at Kahoku site in 2008.

**Table 1:** Determined values of *α*_e_ for each site in Japan, where n is the number of data points for the corresponding year, and R^2^ is the determination coefficient.

Site	Year	*α*_e_	n	R^2^
Sapporo	2001	1.05	48	0.60
	2002	1.03	46	0.68
	2003	1.05	51	0.55
	All years combined	1.04	145	0.61
Kawagoe	1997	1.02	35	0.72
	1998	1.03	25	0.75
	1999	1.04	41	0.90
	2000	1.10	19	0.98
	2001	0.98	35	0.72
	All years combined	1.03	155	0.81
Fujiyoshida	2000	0.99	29	0.81
	2001	1.00	39	0.70
	2002	1.00	37	0.79
	2003	1.04	22	0.89
	2004	1.05	25	0.65
	2005	1.04	29	0.87
	2006	1.03	14	0.85
	2007	1.02	39	0.90
	2008	1.05	25	0.91
	All years combined	1.02	259	0.82
Kahoku	2007	0.95	62	0.89
	2008	0.94	54	0.85
	All years combined	0.95	116	0.87

**Figure 1:**
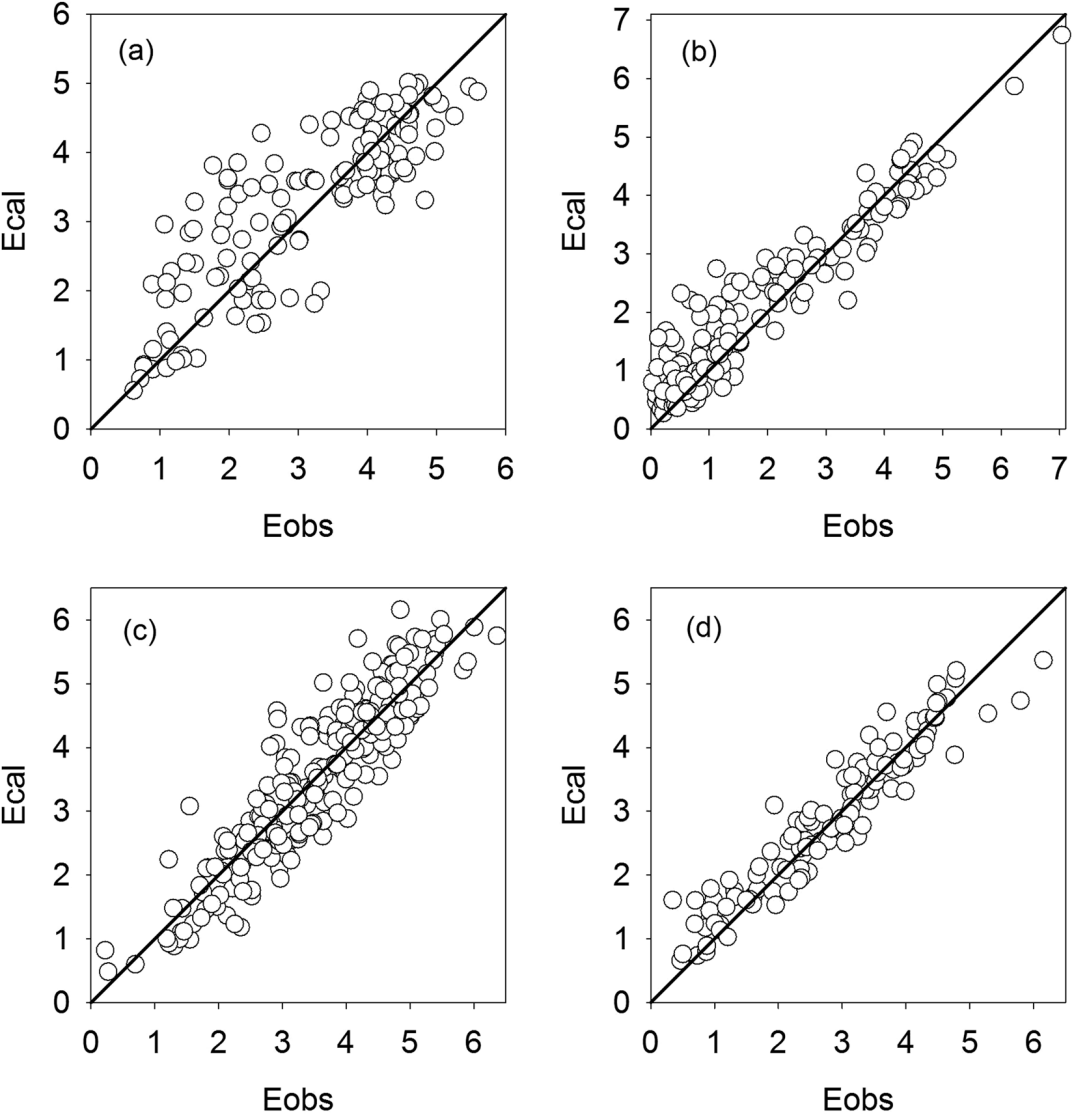
Fit between calculated (cal) and measured (obs) daily mean evaporation (E, mm day^−1^) for (**a**) Sapporo, (**b**) Kawagoe, (**c**) Fujiyoshida and (**d**) Kahoku site in Japan. Data are pooled across years. Full line is the 1:1 line of perfect fit.

As an illustration of the values of *α*_e_, Fig. 1 depicts the goodness-of-fit between the measured and calculated E, pooled for all years at each site. It can be seen that the scatter points were distributed well around the 1:1 line, suggesting that the calculated E was in good agreement with the measured E. The correlation coefficient (R) was 0.78, 0.92, 0.91 and 0.94, for Sapporo, Kawagoe, Fujiyoshida and Kahoku site, respectively, whereas its corresponding absolute bias was 4%, 10%, −0.05%, and 3%. In total, the mean bias was around 4%.

### Evaluation of the new complementary relationship

**Figure 2:**
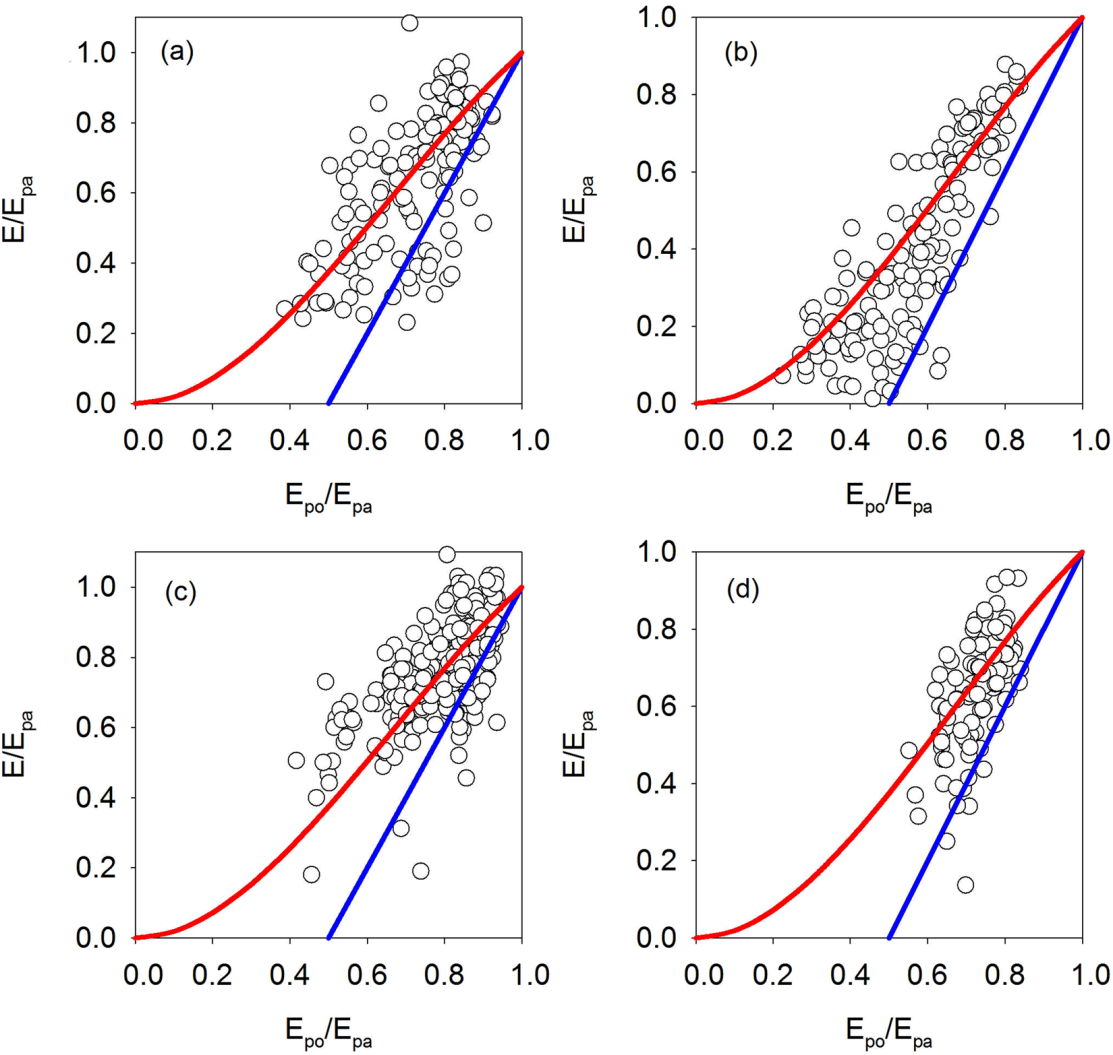
Values of E/E_pa_ as a function of E_po_/E_pa_ at (**a**) Sapporo, (**b**) Kawagoe, (**c**) Fujiyoshida and (**d**) Kahoku site in Japan. Data are pooled across years. Red curves represent the new nonlinear complementary relationship by Brutsaert^11^ and blue curves represent the original complementary relationship by Bouchet^2^. E, E_po_ and E_pa_ are actual, potential and apparent potential evaporation, respectively.

Figure 2 shows the variation of E/E_pa_ as a function of E_po_/E_pa_ calculated with the new complementary relationship (Equation 2) given by Brutsaeart^11^ and with the original relationship (Equation 1) given by Bouchet^2^. It can be seen from this figure that for all the sites, the moisture index E/E_pa_ increased with increasing scaled potential evaporation (E_po_/E_pa_), and the scatter points were distributed closer to the red than to the blue line, indicating a better performance of the new complementary relationship (red line) developed by Brutsaeart^11^ compared to the original linear relationship (blue line) proposed by Bouchet^2^. Furthermore, the discrepancy between the two methods, i.e. lines, increased as the ratio E_po_/E_pa_ decreased. This further pointed on better performance of the new complementary relationship under dry condition (low E_po_/E_pa_ values).

**Figure 3:**
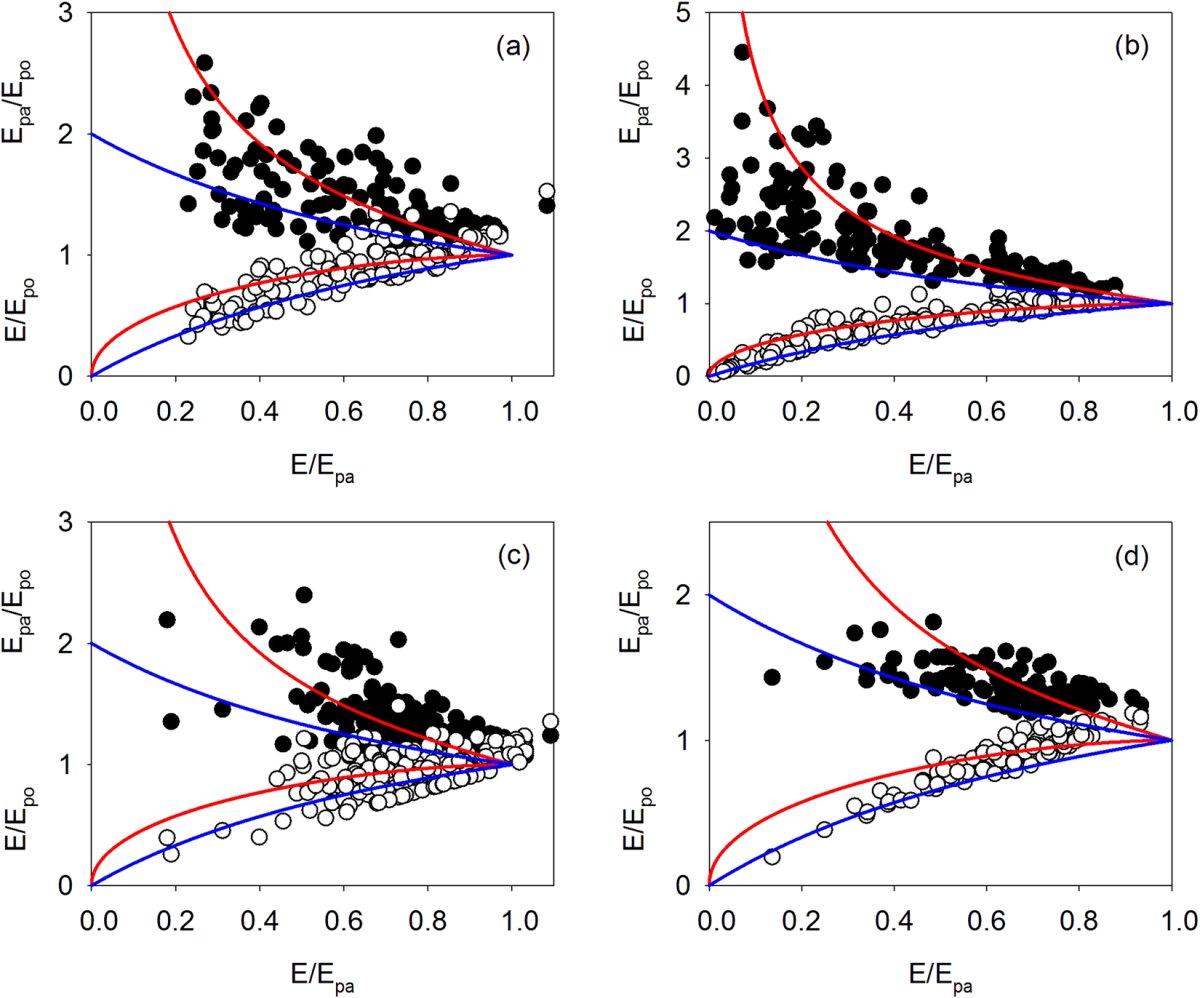
Scaled apparent potential evaporation (E_pa_/E_po_) and scaled actual evaporation (E/E_po_), as a function of the moisture index (E/E_pa_) for (**a**) Sapporo, (**b**) Kawagoe, (**c**) Fujiyoshida and (**d**) Kahoku site in Japan. Data are pooled across years. The red curves represent the new nonlinear complementary relationship by Brutsaert^11^, and the blue curves represent the original complementary relationship by Bouchet^2^. E, E_po_ and E_pa_ are actual, potential and apparent potential evaporation, respectively.

The variation of E_pa_/E_po_ and E/E_po_ as a function of E/E_pa_ calculated with the new complementary relationship (Equation 2) given by Brutsaeart^11^ and with the original relationship (Equation 1) given by Bouchet^2^ are plotted in Fig. 3. It can be seen that E_pa_/E_po_ decreased with increasing E/E_pa_, whereas E/E_po_ actually increased with increasing E/E_pa_. This is because both E_po_ and E are increasing to values close to E_pa_ when the surface wetness condition varies from dry to wet. Further biophysical explanation can be obtained using the boundary limitations described by Brutsaert^11^. It is also evident from the scatter plots on Fig. 3 that the relationship between E_pa_/E_po_ and E/E_po_ is a markedly asymmetrical, and that the new complementary relationship (red line) developed by Brutsaeart^11^ performs better than the original linear relationship (blue line) proposed by Bouchet^2^ in depicting the variations of E_pa_/E_po_ and E/E_po_ with E/E_pa_.

## Discussion

The linear complementary relationship between E, E_po_ and E_pa_ shown in Equation 1 is controversial among scientists due to the lack of sufficient evidence for the inherent assumptions of the Bouchet’s theory. The latest attempt for its improvement is the one proposed by Brutsaert^11^ in a nonlinear form, and estimates of evaporation using this relation have been very few but encouraging. Zhang^13^ used the new improved method to estimate evaporation from different vegetation and climatic conditions across Australia, achieving reasonable fit against measurements with R^2^ ranging from 0.46 to 0.85. In the present study, the method yielded accurate daily evaporation estimates (Fig. 1), with R^2^ ranging from 0.55 to 0.91 (Table 1) when the apparent potential evaporation or evaporative demand was determined with the Penman equation. Moreover, the complementary relationship between E_pa_/E_po_ and E/E_po_ was very similar and markedly asymmetrical across all four sites (Fig. 3), providing sound evidence to support the assumptions underlying Equation 2. The asymmetry itself appears to be a direct result of the boundary conditions used in its derivation.

The determined *α*_e_ values were generally close for Sapporo, Kawagoe and Fujiyoshida sties, but lower at Kahoku site (Table 1). This difference can be explained by the combined effects of vegetation type and nature of surface such as slope and microclimate, as noted by Brutsaert^11^. As for the Kahoku site, the forest type is mainly coniferous, which has relatively lower evapotranspiration. Former study had also reported the low value of *α*_e_ (0.72) for the coniferous forest^15^. Terrain type at the site is rolling terrain, which might also reduce the *α*_e_ value^12^. In addition, the relative lower net radiation and wind speed could also reduce the *α*_e_ value. For example, the mean net radiation and mean wind speed (2 m) at the site is 141 W m^−2^ and 0.4 m s^−1^, respectively. The determined *α*_e_ values were overall consistent with those reported in the literatures. Most recently, Brutsaert^12^ used the new nonlinear complimentary method for trees, small crops and grassy vegetation in the Loess Plateau in China and obtained *α*_e_ values of about 1.02. Zhang^13^ utilized different methods in estimating apparent evaporation for various vegetation covers in Australia and estimated *α*_e_ values between 1.00 and 1.19. Liu^14^ calculated *α*_e_ values between 0.95 and 1.30 for various land covers across eastern China at regional scale. Based on the original linear complementary method of Bouchet^2^, mean *α*_e_ values for Sweden, eastern China, and Cypus were reported as 1.18, 1.00, and 1.04, respectively^16^. Weekly *α*_e_ values calculated with the original method and reported by Yang^17^ varied between 1.00 to 1.20 in summer monsoon season, and between 1.20 to 1.70 in winter monsoon season, for a hilly evergreen forest in northern Thailand. For the North China Plain, the *α*_e_ values calculated with the original method ranged from 0.80 to 1.50 in summer monsoon season, and ranged from 1.20 to 2.20 in winter monsoon season^17^. Apparently, *α*_e_ in the new complementary method is largely different from the original meaning under truly potential conditions. However, as described by Brutsaert^12^ and Zhang^13^, *α*_e_ values calculated by the new complementary method were close to those determined by the original Priestley–Taylor equation. For example, mean *α*_e_ value of 1.05 was reported for a Douglas fir forest^18^ and of 0.72 for different types of coniferous forest^15^. This study further confirmed this tendency according to the results in Table 1.

## Conclusion

This study evaluated the new nonlinear complementary relationship proposed by Brutsaert^11^ for estimation of forests evaporation in Japan. The only unknown parameter in the relationship, *α*_e_, was determined for the first time for various forest vegetation of north to south Japan. The mean value of *α*_e_ was 1.01, ranging from 0.94 to 1.10. The calculated forests evaporation showed a good result with the measured values, with an R^2^ of 0.78 and a bias of 4% on average. Moreover, the new nonlinear relationship performed better than the original linear relationship of Bouchet^2^ in describing the relation between E/E_pa_ and E_po_/E_pa_, and also in depicting the asymmetry variation between E_pa_/E_po_ and E/E_po_. Overall, the results of this study lend credibility to the evaporation prediction skill of the new nonlinear complementary relation for forest land cover in Japan, making it a reasonable forecasting tool for this region that can be also tested and verified for other land covers and regions.

## Materials and Methods

### The new nonlinear complementary model

Bouchet, based on the two boundary conditions, arrived at the following linear complementary relationship between actual evaporation (E), potential evaporation (E_po_), and apparent potential evaporation (E_pa_)^2^:

1 $$E=2{E}_{{\rm{p}}{\rm{o}}}-{E}_{{\rm{p}}{\rm{a}}}$$

Equation 1 says that an increase of water availability at the surface, e.g. by irrigation, is concomitant with the reverse process, and that E_pa_ decreases as E increases. Thus, potential evaporation measured over a region becomes both the result and cause of actual evaporation measured over the same region^19^.

Recently, Brutsaert used four boundary conditions to modify the linear complementary relationship between E, E_po_ and E_pa_ (Equation 1) with a cubic polynomial model^11^:

2 $$E={(\frac{{E}_{{\rm{po}}}}{{E}_{{\rm{pa}}}})}^{2}(2{E}_{{\rm{pa}}}-{E}_{{\rm{po}}})$$

In this study, E_po_ is approximated by Priestley–Taylor equation^4^:

3 $${E}_{po}={\alpha }_{{\rm{e}}}\frac{{\rm{\Delta }}}{{\rm{\Delta }}+\gamma }({R}_{n}-G)$$

and E_pa_ is estimated using Penman equation^7^:

4 $${E}_{pa}=\frac{{\rm{\Delta }}}{{\rm{\Delta }}+\gamma }({R}_{n}-G)+\frac{\gamma }{{\rm{\Delta }}+\gamma }{f}_{e}({u}_{1})({e}_{2}^{\ast }-{e}_{2})$$

where *α*_e_ is a parameter of the Priestley–Taylor equation, Δ is the slope of the saturation vapor pressure curve, *γ* is the psychrometric constant, *R*_*n*_ is net radiation, *G* is the surface ground heat flux, ${e}_{2}^{\ast }$ and *e*_2_ are saturation vapor pressure and actual vapor pressure at a height above the surface, respectively. *f*_*e*_(*u*_1_) is the wind function. As noted by Zhang^13^, the choice of wind function has low effect on the calculated E, thus, a simple wind function was adopted in the present study^6^:

5 $${f}_{e}({u}_{2})=0.35(1+0.54{u}_{2})$$

where *u*_2_ is wind speed at the height of 2 m that can be obtained by the wind speed (*u*_*z*_) measured at a height (z) using the equation by Brutsaert^20^:

6 $${u}_{2}={u}_{z}{(\frac{2}{z})}^{\frac{1}{7}}$$

### Study sites and data

**Figure 4:**
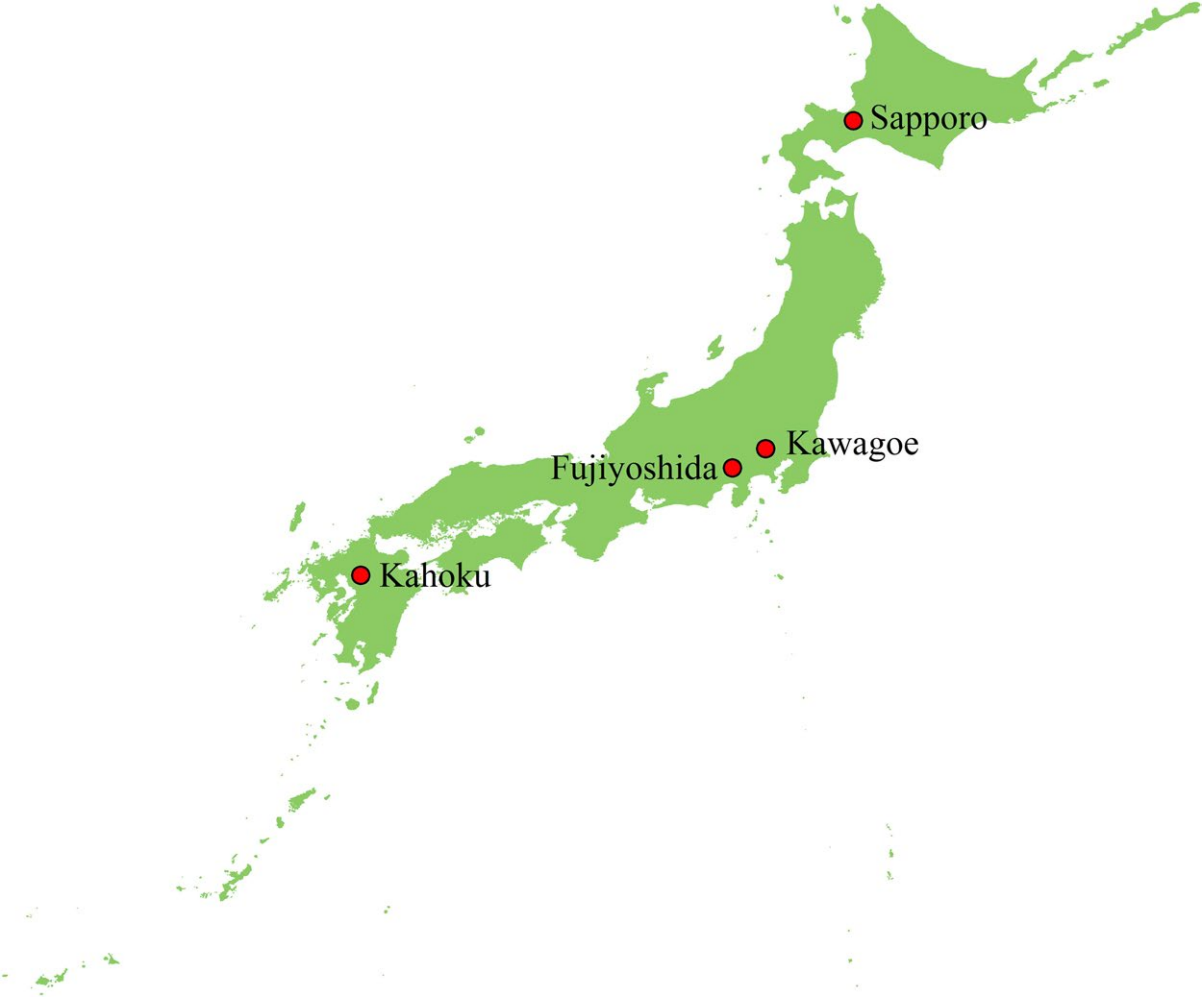
A map showing the study sites in Japan. This map was created by ArcMap (version: 10.4.0.5524; link: https://www.esri.com).

This study was conducted on four forest sites located from north to south across Japan, namely, Sapporo, Kawagoe, Fujiyoshida, and Kahoku (Fig. 4). The dominant vegetation cover is deciduous broadleaf forest, deciduous broadleaf forest, secondary natural evergreen needleleaf forest and evergreen coniferous forest, for Sapporo, Kawagoe, Fujiyoshida, and Kahoku, respectively. The climate across Japan is temperate, but it varies with a north-south gradient, being cool at Sapporo and warm at Kahoku. All sites belong to the FluxNet network of the Forestry and Forest Products Research Institute of Japan (http://www2.ffpri.affrc.go.jp/labs/flux/). More detailed description of the sites including the location, elevation, and the data period used in the study is given in Table 2. At each site, half-hourly precipitation, air temperature, relatively humidity, wind speed, net radiation, sensible and latent heat, and soil heat flux were recorded (Table 3). More detailed information about the measurements at the sites can be found elsewhere^21–24^.

**Table 2:** Detailed information of the four flux sites used in this study.

Site	Location	Forest type	Elevation (m)	Air temperature (°C)	Precipitation (mm)	Period (years)
Sapporo	42.9868 N, 141.3853E	deciduous broadleaf	182	7	980	2001–2003
Kawagoe	35.8725 N, 139.4869E	deciduous broadleaf	26	15	1300	1997–2001
Fujiyoshida	35.45454 N, 138.76225E	secondary natural evergreen needleleaf	1030	9.5	1955	2000–2008
Kahoku	33.137 N, 130.7095E	evergreen coniferous	165	15.3	2138	2007–2008

**Table 3:** Measurement height or depth (m) for the variables at each study site in Japan.

Site	Precipitation	Air temperature	Relative humidity	Wind speed	Net radiation	Sensible heat	Latent heat	Soil heat flux
Sapporo	1.8	41	41	41	41	28.5	—	0.02
Kawagoe	0.6	21	21	21	25	20	—	0.02
Fujiyoshida	1.0	23	23	32	32	25	—	0.02
Kahoku	1.5	42	42	51	47	51	51	0.05

For each site, E, E_po_ and E_pa_ were estimated according to Equations 2, 3 and 4, respectively, as presented above. The unknown coefficient *α*_*e*_ was determined by regression of Equation 2 using trial and error such that the slope of the regression through the origin equals unity^12^. E was also estimated from the eddy covariance measurements of sensible and latent heat fluxes using the energy budget closure. This approach requires that the sum of the measured latent heat and sensible heat fluxes equals to all other energy sinks and sources (i.e., R_n_ − G). As summarized by Twine^25^, two methods can be used for energy budget closure, namely, the ‘residual closure’ and the ‘Bowen-ratio closure’. For Sapporo, Kawagoe and Fujiyoshida sites with available only sensible heat measurements, the ‘residual closure’ method was used. As it is known that eddy covariance measurements underestimate the sensible heat within around 30%^25^, an increase of 15% was added to the sensible heat measurements at these sites. For Kahoku site, both latent heat and sensible heat fluxes were measured and the ‘Bowen-ratio closure’ was used assuming it is preserved over the entire range of the turbulence spectrum^13,25^.

Considering the effect of atmospheric stability, the measured half-hourly data were averaged to daily values. Hence, there should be 48 records with the half-hourly measurement frequency every day for each site. However, the entire day was excluded from the analysis if two or more records were missing for a day. Further, data pre-processing ensures sound relationships between the variables in the analysis, and two data requirement steps were conducted^12^: 1) only data measured on days without rain were included, and 2) data were excluded if the wind speed was smaller than 0.2 m s^−1^, or the net radiation was less than 20 W m^−2^, or the calculated heat was less than 0 W m^−2^, or the sensible heat was less than 30 W m^−2^, or the air temperature was below freezing.
